# The genetics of phenotypic plasticity. XV. Genetic assimilation, the Baldwin effect, and evolutionary rescue

**DOI:** 10.1002/ece3.3429

**Published:** 2017-09-18

**Authors:** Samuel M. Scheiner, Michael Barfield, Robert D. Holt

**Affiliations:** ^1^ Division of Environmental Biology National Science Foundation Arlington VA USA; ^2^ Department of Biology University of Florida Gainesville FL USA

**Keywords:** Baldwin effect, climate change, evolutionary rescue, genetic assimilation, model, phenotypic plasticity

## Abstract

We used an individual‐based simulation model to examine the role of phenotypic plasticity on persistence and adaptation to two patterns of environmental variation, a single, abrupt step change and continual, linear change. Our model tested the assumptions and predictions of the theory of genetic assimilation, explored the evolutionary dynamics of the Baldwin effect, and provided expectations for the evolutionary response to climate change. We found that genetic assimilation as originally postulated is not likely to occur because the replacement of plasticity by fixed genetic effects takes much longer than the environment is likely to remain stable. On the other hand, trait plasticity as an enhancement to continual evolutionary change may be an important evolutionary mechanism as long as plasticity has little or no costs. Whether or not plasticity helps or hinders evolutionary rescue following a step change in the environment depends on whether plasticity is costly. For linear environmental change, noncostly plasticity always decreases extinction rates, while costly plasticity can create a fitness drag and increase the chance of extinction. Thus, with changing climates plasticity can enhance adaptation and prevent extinction under some conditions, but not others.

## 1 INTRODUCTION

A ubiquitous feature of the environment is its heterogeneity, and change in the environment over time can result in a shift in an organism's optimal trait value. Environmental change can take many forms, such as variability around a central value without any directional trend, a one‐time, abrupt shift in the environment, or a continual, directional change in the central value that may or may not also include nondirectional change around that central value. In this study, we focus on two simple patterns—a single, abrupt shift and continual, linear change—that for convenience we refer to as “step change” and “continual change.” Although environmental heterogeneity in nature is generally more complex, our exploration through simulation models considers each of these simple types of change alone so as to isolate salient patterns of adaptation. While environmental heterogeneity has always been a factor in evolution, our analyzes take on increased urgency in the face of large, human‐induced environmental shifts, including global warming, which continues to move our world into novel patterns of selection and demographic risk for many species.

Changes in the mean traits exhibited by a population in response to a shift in optimal trait values can include two components: changes in genes that have a fixed effect on phenotype and a plastic change in the phenotype. Often these are posited as alternative responses, but they can occur together (Hoffmann & Sgrò, 2011; Merilä & Hendry, 2014; Valladares, Wright, Lasso, Kitajima, & Pearcy, 2000; Visser, 2008). Moreover, phenotypic plasticity itself is an evolved and evolving response. In this study, we ask how the evolutionary response to environmental change is affected by the presence and evolution of phenotypic plasticity. We show that phenotypic plasticity can play an important role in that response, but the effect is strongly tempered by the patterning of that change and whether or not plasticity has a fitness cost or limitation (DeWitt, Sih, & Wilson, 1998). A cost is a factor that decreases the fitness of an individual even when the trait matches the optimum. A limitation is a factor that prevents an individual from matching that optimum, which in our model takes the form of developmental noise, that is genetically linked to phenotypic plasticity.

Our modeling efforts were guided by three prior theories; our aim is to formalize two earlier verbal theories of the role of phenotypic plasticity in evolution and to unite them with a quantitative theory of population responses to environmental change. Of the two plasticity theories, the older is that of Baldwin (1896, see also Morgan, 1896; Osborn, 1896). To use modern parlance, Baldwin posited that trait plasticity would allow continual improvement in organismal adaptation that would occur faster than if adaptation occurred strictly through the evolution of nonplastic genetic elements. His theory was based on an assumption that a population is always subject to continual directional selection for an increased trait value without regard specifically to changes in the external environment. We retain that assumption, but rework it into one by which the environment is continually changing in a directional fashion, resulting in a continual, directional selection on trait values. Translating a pre‐Mendelian verbal model into today's understanding is always fraught; we translate Baldwin into saying that either there existed some distant trait optimum that the population was evolving toward, or that competition within the population was always moving the optimum toward the most extreme value within the population, as in models of runaway sexual selection. Our model takes those ideas and applies them to a scenario not envisaged by Baldwin, namely a fitness change driven by changes in the external environment. Only two formal models exist of the effects of plasticity on adaptation to a continually changing optimum, those of Chevin, Lande, and Mace (2010) and Nunney (2015). In both of those models, the existence of phenotypic plasticity increased population survival in the face of continual environmental change. However, in both trait plasticity is nonevolving. Our model expands on those efforts by allowing plasticity to also evolve.

The second is Waddington's (1942, 1953) theory of genetic assimilation. He posited that in response to a step change in the environment, trait plasticity would allow an organism to survive until the evolution of nonplastic genetic elements replaced the plastic response. West‐Eberhard's (2003) theory of genetic accommodation also considered evolution following a step change in the environment, expanding on Waddington's theory in two ways (Crispo, 2007). First, it allowed plasticity to remain or increase as in Baldwin's original theory. In addition, it expanded the notion of the environment of a gene to include the rest of the genome; a genetic change that affected one trait could induce a plastic change in another trait that would then be subject to selection. We focus here on Waddington's version of the theory, although our conclusions also hold for West‐Eberhardt's expanded version.

No formal model of Waddington's theory has ever been developed. Lande (2009, 2015) modeled evolution following a step change with plasticity. Although he stated that his was a model of genetic assimilation, it did not quite mirror Waddington's verbal theory. In Waddington's theory, the environment is homogeneous before and after the step change; in Lande's model the environment before and after is subject to small‐scale temporal variation. As we show, this difference is critical to the evolutionary outcome. In Chevin and Lande (2010), the model assumed a constant environment as per Waddington, but also always included a cost of plasticity, which was not included in Waddington's verbal theory. As we show, such a cost is also critical to the evolutionary outcome. We formalize Waddington's original theory because it is very widely cited and the notion of genetic assimilation is deeply embedded in the literature (Crispo, 2007); using Google Scholar, we found over 400 articles in 2015 alone that mention the concept and a total of 576 citations of Waddington (1953). It is important that such a widely held theory be formally tested. In doing so, we shed light on related models (Chevin & Lande, 2010; Lande, 2009) and the conditions under which they are likely to hold.

The third is Gomulkiewicz and Holt's (1995) theory of evolutionary rescue. Evolutionary rescue occurs following a change in the environment when a population, that is, declining in numbers because of maladaptation evolves sufficiently so that population numbers increase again and the population recovers (the term can also be used to denote persistence by selection, i.e., sufficiently rapid that population decline does not occur). Neither genetic assimilation nor the Baldwin effect takes into account ecological dynamics. Such dynamics can constrain the conditions under which trait plasticity can play a role in evolutionary dynamics. Conversely, the presence of trait plasticity can change ecological dynamics and allows adaptation in conditions under which it otherwise would not occur. Our simulations explore that interaction. Chevin and Lande (2010) demonstrated that phenotypic plasticity can enhance evolutionary rescue. Our individual‐based simulation model is complementary to their quantitative‐genetic analytic model, allowing us to look at the probability of extinction and to confirm their results using different genetic assumptions. We also extend those results by additionally considering the effects of limitations on plasticity and continual environmental change.

### 1.1 Modeling goals

Our modeling efforts had three sets of goals: (1) formalize the assumptions and test the verbal predictions of the theory of genetic assimilation, (2) explore the evolutionary dynamics of the Baldwin effect, and (3) indicate how those models might inform our expectations for the evolutionary response to climate change, including evolutionary rescue (or not), and what we would need to measure to use that information for management purposes. Considering the first goal, genetic assimilation, and related theories make the following assumptions. A population in a stable environment has some sort of unexpressed plastic capacity in trait expression. That trait plasticity gets expressed following a step change in the environment to a new stable environment. Following that change, the plastic capacity in trait expression is selected to disappear. We tested these predictions with a series of comparisons: a starting population with no initial mean plasticity versus one with initial plasticity, and plasticity being costly or having limitations versus having no cost or limitations, each for a range of magnitudes of the step change. The test consists of examining: the probability of the population surviving the change, the rate of the evolutionary response, whether the evolutionary response is primarily due to changes in plastic or nonplastic loci, and the pattern of change in the genetic variation of those loci.

To address the second goal, in addition to exploring the effects of a step change in the environment, we also examined the effects of continual change, the Baldwin effect. The same types of conditions were compared, except that differences in the rate of environmental change were considered instead of differences in the magnitude of the step change. We only explored the effects of a cost of plasticity, not limitations. The latter will be included in a future study. For response variables, we also consider the extent to which the mean phenotype of the population lagged behind the optimal phenotype.

Our two scenarios—a stable environment before and after a step change and steady continual change—are meant to bracket real‐world patterns of environmental change. Long‐term, directional environmental change is almost never unidirectional. It always consists of some average trend imposed upon change that varies in rate and direction. The environment is always varying even if there is no directional change. And a step change‐like response could occur during a directional change in the environment if there was a period of relatively slow change followed by a sudden, rapid change and then additional slow change.

Thus with regard to our third goal, predicting and managing the effects of climate change, our models do not make specific predictions about any single system. Rather, they indicate when phenotypic plasticity might or might not play an important role in nature's response to coming changes. They tell us when it might behoove us to measure trait plasticity and the costs of that plasticity, given expectations about the rate and pattern of environmental change. Armed with that information, more targeted models can then be developed (Reed, Waples, Schindler, Hard, & Kinnison, 2010).

## 2 THE MODEL

We used an individual‐based model (implemented in Fortran 90) to simulate the effects of phenotypic plasticity on environmental rescue in response to an environmental change that happens either once (a step change) or continuously (a linear change). The model is based on that of Bürger and Lynch (1995), to which we have added genetically determined phenotypic plasticity. In our model, individuals are diploid, hermaphroditic, mate once and die after reproduction, with nonoverlapping generations. Each individual has a trait (phenotype *T*) that determines its juvenile survival (which is when selection takes place).

### 2.1 Determining the phenotype

In our model, an organism's total phenotype is the sum of three components: contributions to the phenotype due to nonplastic loci, contributions due to plastic loci that interact with the environment, and a random component. The phenotype *T*_*ij*_ of the *i*th individual developing in generation *j* is given by:

(1) $$T_{ij}=\sum_{k=1,2n}N_{ijk}+\;E_{j}b\sum_{k=1,2m}P_{ijk}+\;z_{ij},$$

where *N*_*ijk*_ are allelic values at the *n* nonplastic loci, and *z*_*ij*_ is a zero‐mean, unit‐variance independent Gaussian random deviate (the random component of phenotype); these are the components of the original model of Bürger and Lynch (1995). The middle term is the contribution of plasticity to the phenotype, where *P*_*ijk*_ are allelic values at the *m* plastic loci of this individual, and *E*_*j*_ is the environment at the time of trait development in generation *j*. For a clone of genetically identical individuals, taking the expected value of Equation (1) gives *E*[*T*_*ij*_] = *a *+ *dE*_*j*_, which is a linear reaction norm (mean phenotype is a linear function of the environment) with an intercept of *a* (the sum of nonplastic alleles) and a slope (*d*) equal to the product of parameter *b* and the sum of plastic allelic values. The plasticity parameter (*b*) determines the magnitude of the plastic response by the phenotype for a given genetic value, scaling the phenotypic variation due to plasticity relative to the variation due to the nonplastic alleles. In the starting environment (until generation = 0) the value of *E*_*j*_ = 0, so that the phenotypic value is due only to the nonplastic alleles.

### 2.2 Selection

Selection occurred during survival from juvenile to adult, which had a probability (*W*_*ij*_ for the *i*th individual in generation *j*) that was a Gaussian function of the difference between the individual's phenotype and an optimum phenotype for its environment (*T*_*opt,j*_):

(2) $$W_{ij}=\text{exp}\left\{-\frac{1}{2}{\left(\frac{T_{ij}-T_{opt,j}}{\omega }\right)}^{2}\right\},$$

where ω determined the strength of selection on the phenotype (a lower value being stronger selection). Fitness is the product of *W*_*ij*_ and fecundity. The survival probability was 1 for an individual with trait *T*_*ij*_ equal to the optimum *T*_*opt,j*_, and decreased as the difference between *T*_*ij*_ and *T*_*opt,j*_ increased. The effective selection was stabilizing when the population mean trait value was near the optimum, and directional when the mean was far from the optimum. We scaled the optimum phenotype so that *T*_*opt,j*_ = *E*_*j*_, assuming that the environments at the times of development and selection in any generation were the same (although they could change across generations).

### 2.3 Costs and limitations

To this basic model we added either a cost of or a limitation to plasticity. To model a cost, we modified the survival function by allowing stabilizing selection on the sum of the plasticity alleles around 0 (i.e., any departure from 0 lowers fitness) as in Chevin and Lande (2010). Adding a cost of plasticity, the survival probability became:

(3) $$W_{ij}\;=\;\text{exp}\left\{-\frac{1}{2}{\left(\frac{T_{ij}-T_{opt,j}}{\omega }\right)}^{2}-\frac{c}{2}{\left(\frac{\sum_{k}P_{ijk}}{\omega_{P}}\right)}^{2}\right\},$$

where *c* is 1 if there is a cost of plasticity and 0 if not, and ω_*P*_ determines (inversely) the cost of plasticity. This cost of plasticity was independent of allelic expression; even when plasticity was not expressed (i.e., *E*_*j*_ = 0), the plasticity loci still had an effect on fitness. An example of such an independent cost would be maintaining additional cellular or organismal machinery needed to translate an environmental signal into a phenotypic change. Alternative forms of cost could be either a fixed cost, given the existence of any nonzero plastic alleles, or a cost that scales with the phenotypic expression of the plastic alleles, not just their genotypic values. We consider the implications of these alternatives in the Discussion.

To model a limitation, we introduced developmental noise in the effect of plasticity on the phenotype by adding a second variable that randomly altered the phenotype. The magnitude of this noise was linked to the expression of the plasticity alleles:

(4) $$T_{ij}\;=\;\sum_{k=1,2n}N_{ijk}+\left(1+y_{ij}s\right)E_{j}b\sum_{k=1,2m}P_{ijk}+z_{ij}.$$

Here, *y*_*ij*_ is a zero‐mean, unit‐variance independent Gaussian random deviate and *s* scales the amount of developmental noise. In other words, plasticity was pleiotropically related to a portion of the developmental noise. This term could include random differences in the microenvironments in which individuals develop or in effects of the environment on expression of the plastic loci, and random variation in the processes initiated or influenced by the plastic loci. Throughout the rest of the paper, we refer to this portion as “developmental noise” recognizing that the *z*_*ij*_ term is accounting for the other portion (i.e., *z*_*ij*_ includes random developmental variation not related to expression of the plastic loci). This new term operationalizes Waddington's (1942) suggestion that selection will replace plasticity with a fixed response because nonplastic development will result in a more accurate phenotype (i.e., the new term represents error in the contribution of the plastic alleles to the phenotype, while there is no error for the nonplastic alleles). Fitness was determined using Equation (2) because limitations were modeled with no cost of plasticity.

### 2.4 Reproduction and mutation

Reproduction followed selection. Density was regulated by limiting the number of matings to a value *K*. If there were fewer than *K* adults, then all adults mated as a female; if there were more than *K*,* K* were chosen at random (without replacement) to act as females. (This mating system differs from that in Bürger and Lynch (1995), which includes a weak Allee effect.) Each mating female was paired with an adult randomly selected with replacement to act as a male; this could be the same individual as the female, that is selfing was allowed. (However, as mating is random, the degree of selfing depends only on population size and is only significant when the population size is small, which occurred only for some of the step change simulations and only for a brief period.). Each mated pair produced *f* offspring.

During reproduction, there was free recombination. Allelic values could take on any real value. Each offspring haplotype mutated with probability (*n*+*m*)μ, μ being the per‐locus mutation rate. If a mutation occurred, a random locus was selected, and a zero‐mean Gaussian with variance α^2^ was added to the previous allelic value; the mutation rate and variance for plastic and nonplastic alleles were the same. After the alleles of each offspring were determined, the random component of its phenotype was chosen. Because the plasticity parameter (*b*) determines how genetic variation gets translated into phenotypic variation, a greater value means that the same mutational change has a greater phenotypic effect. That is, the plasticity parameter is proportional to the rate of new plastically determined phenotypic variation.

### 2.5 Initial conditions

For all simulations described below, a population was initiated with *K* adults with random nonplastic alleles that gave the expected steady‐state genetic variance (i.e., the variance among individuals of the sum of the allelic values, Σ*N*_*ijk*_); we used the stochastic house‐of‐cards variance in Bürger and Lynch (1995). There was a 1,000 generation equilibration period, with the phenotypic optimum held constant at 0, to allow the population to reach mutation‐selection‐drift equilibrium. This is the same protocol we used in previous work (e.g., Holt, Gomulkiewicz, & Barfield, 2003). In simulations with no initial mean plasticity, plastic alleles were initialized in the same way as nonplastic alleles, with one modification. Because the plasticity alleles have no effect on phenotype during the equilibration period, their values would be unconstrained were plasticity costless. Therefore, we set *c *=* *1 during the equilibration period, even when there was no cost to plasticity after equilibration, so that the genetic variance of the plastic alleles was comparable to that of the nonplastic alleles at the end of equilibration.

We investigated two different starting conditions for plasticity, a mean relative plasticity of the population at 0.0 and at 0.2. Because the environment was constant during the equilibration period, there was no benefit to plasticity and the cost of plasticity created stabilizing selection on those loci resulting in a mean plasticity of approximately 0.0. For simulations with a nonzero (0.2) initial mean plasticity, we used a procedure that was equivalent to simply adding to each plastic allele at the end of equilibration the constant amount [0.2/(2*mb*)] needed to increase the mean relative plasticity to the desired value. This procedure resulted in all simulations having the same initial genetic variance in plasticity alleles.

### 2.6 Environmental change

After the equilibration period, for the step change the optimum phenotype was abruptly altered in a single generation by a positive value. For continual change, the optimum phenotype increased by a fixed increment each generation, resulting in a linear change over time. (Throughout the manuscript, “initial” refers to the time the environment changed or started to change following the equilibration period, and all time plots start then.) For a step change, the amount of change varied from 2 to 4 times the width of the fitness function (ω); for continual change, the total amount of change varied from 20 to 500 times the width of the fitness function, depending on the rate of change. For a step change, the simulation was continued for 500 generations; for continual change it continued for 1,000 generations. These periods were chosen because (1) for a step change, in 500 generations all populations either went extinct or adapted, and the long‐term trends were apparent in the response parameters for the surviving populations, or (2) for continual change, it is highly unlikely that a continuous, steady change would persist for longer than that period. For continual change, in our model the phenotypic response of a plastic individual will get larger, even if there are no additional genetic changes, because of the change in the environmental inducer. Thus, we assumed that there were no intrinsic morphological, physiological, or developmental limits to the plastic response.

### 2.7 Parameter sets and response variables

Our results are presented in terms of relative phenotype and relative plasticity, which are values of mean phenotype and plasticity normalized to the optimum phenotype. The relative phenotype for a population is therefore its mean phenotype divided by the optimum phenotype *T*_*opt*,*j*_, so that a perfectly adapted population has a relative phenotype of 1.0. Its relative plasticity is the mean plastic component of phenotype [the middle term on the right side of Equation (1)] divided by *T*_*opt*,*j*_, which is a measure of the contribution of the plastic alleles to adaption (one minus the relative plasticity is the contribution due to nonplastic alleles). A population with a relative plasticity of 1.0 can remain at a changing optimal phenotype without any evolution of the nonplastic alleles because the middle term of Equation (1) is then equal to *T*_*opt*,*j*_ = *E*_*j*_.

We examined the effects of a cost of plasticity for both types of environmental change. For each we varied the initial average relative plasticity (0 or 0.2), the plasticity parameter *b*, and the degree of change of the environment. To examine the cost of plasticity, we set *c* to 0 or 1; for simulations with a cost there was no developmental noise (*s *=* *0). When both *b *=* *0 and *c *=* *0, our model matches previous models without plasticity and serve as a baseline for comparison. For parameter values of *b *=* *0 and *c *=* *1, the plasticity loci incur a fitness cost but never affect trait values and serve as an indicator of just the cost effect. For each parameter set, 1,000 replicates were run, and the probability of persistence was the fraction of such populations that were not extinct after a specified period (500 generations for the step change and 1,000 generations for the linear change).

We examined the effects of a limitation on plasticity only for a step change. We varied the developmental noise parameter (*s*), set the plasticity parameter (*b*) to 0.6, and set the cost of plasticity to 0. As above, we varied the initial average relative plasticity (0 or 0.2), the plasticity parameter *b*, and the degree of change of the environment, and ran 1,000 replicates for each parameter set.

For all simulation, the other parameters were as follows: maximum population size *K *=* *256, fecundity *f *=* *4, strength of selection parameter ω^2^ = 1, number of nonplastic and plastic loci *n = m = *10, mutation rate μ = 0.0005 (per haplotype mutation rate of 0.01), and variance of mutation size α^2^ = 0.05. For each parameter set, at each generation we recorded the relative phenotype, relative plasticity, the variance of the phenotype, the genetic variances and covariance of the plastic and nonplastic components of the phenotype (i.e., the sum of the plastic or nonplastic alleles, respectively), and the fraction of populations that survived to that generation. These variables (except the last) were averaged over all populations that survived to the end of the simulation. In the results, we show final values of these quantities and, in some cases, their time courses. As a test of the effects of initial population size, for the step change and no initial mean plasticity, we also performed simulations with and without plasticity costs for *K *=* *64 and *K *=* *1,024, and found very similar patterns to those reported here (results not shown).

## 3 RESULTS

### 3.1 Step change ‐ survival

**Figure 1:**
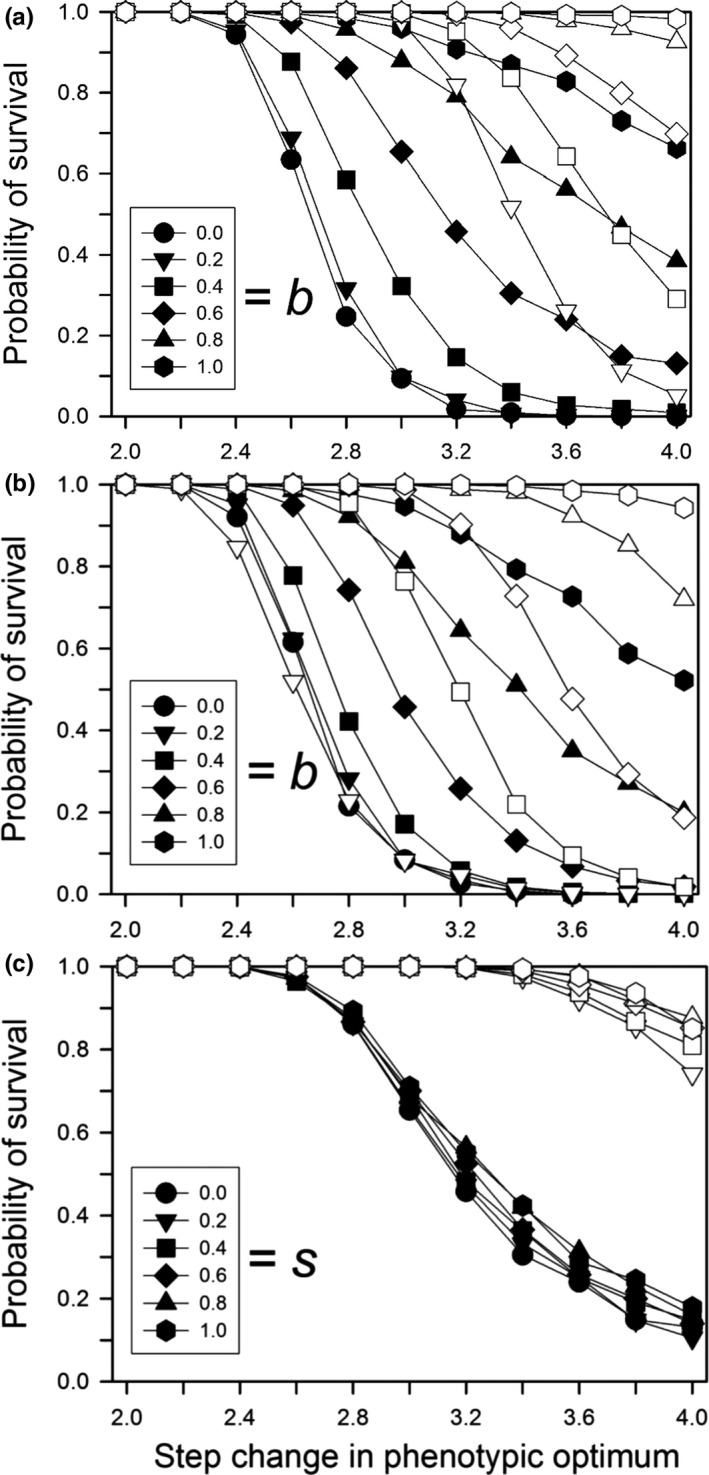
For a step change in the environment, the probability of survival to generation 500 for different expected initial amounts of relative plasticity (0 = solid symbols; 0.2 = open symbols). Other parameters were as follows: *K *=* *256, *f *=* *4, ω = 1, *n = m = *10, μ = 0.0005, α^2^ = 0.05, ω_*P*_ = 1. (a) Without plasticity costs or developmental noise for different plasticity parameters (*b*); (b) with plasticity costs for different plasticity parameters (*b*) without developmental noise; (c) with developmental noise for different amounts of noise (*s*) without plasticity costs (*b *=* *0.6)

**Figure 2:**
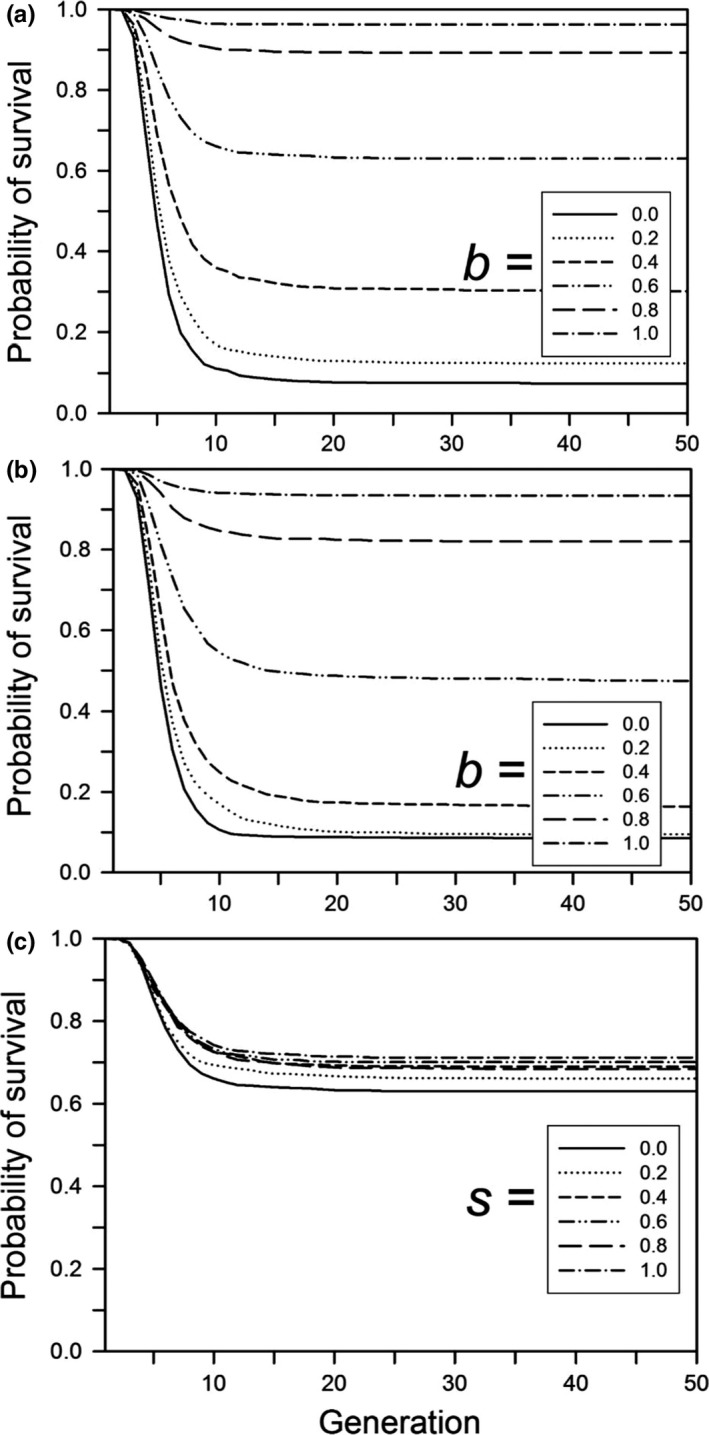
For a step change in the environment, the probability of survival as a function of the time since the step change, for populations that survived for 500 generations. Other parameters were as follows: *K *=* *256, *f *=* *4, ω = 1, *n = m = *10, μ = 0.0005, α^2^ = 0.05, ω_P_ = 1. (a) Without plasticity costs or developmental noise for different plasticity parameters (*b*); (b) with plasticity costs for different plasticity parameters (*b*) without developmental noise; (c) with developmental noise for different amounts of noise (*s*) without plasticity costs (*b *=* *0.6). The step change in the environment was 3.0 units

**Figure 3:**
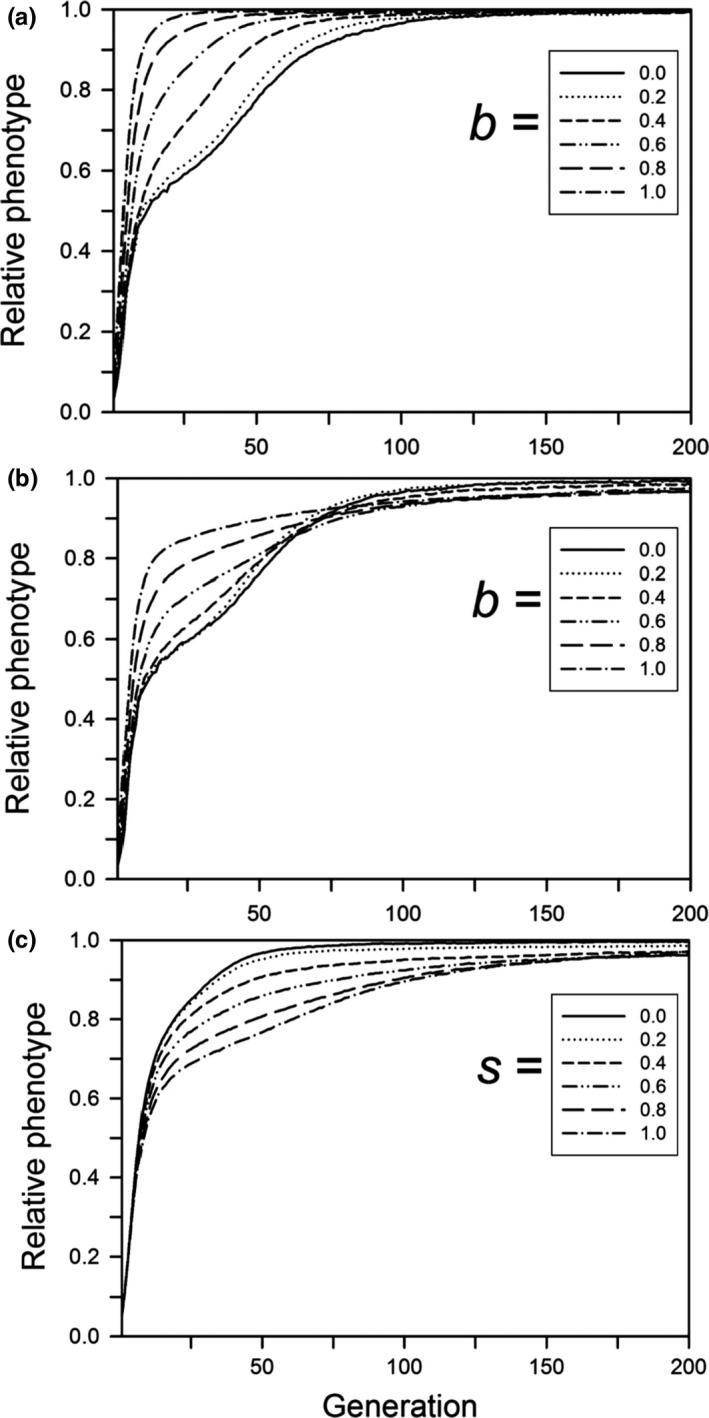
For a step change in the environment, the relative phenotype as a function of the time since the step change, for populations that survived for 500 generations. Other parameters were as follows: *K *=* *256, *f *=* *4, ω = 1, *n = m = *10, μ = 0.0005, α^2^ = 0.05, ω_P_ = 1. (a) Without plasticity costs or developmental noise for different plasticity parameters (*b*); (b) with plasticity costs for different plasticity parameters (*b*) without developmental noise; (c) with developmental noise for different amounts of noise (*s*) without plasticity costs (*b *=* *0.6). The step change in the environment was 3.0 units

Following a step change in the environment, the probability of population survival decreased with the size of the step change, unless the change was small enough that all populations survived or large enough that none survived (Figure 1). If a population survived, adaptation to the new environment occurred quickly (with or without a cost of plasticity; Figures 2a and 3a). Very few populations went extinct after generation 20, indicating that almost all either had adapted by that time (in which case they persisted for the rest of the simulation) or had gone extinct. In other words, if environmental change is quite small, or very large, then plasticity and genetic evolution (and their interplay) are largely irrelevant, in the former case because the population would persist anyway, and in the latter because the demographic costs of the environmental change overwhelm any likely response, so extinction is inevitable.

When plasticity was not costly, for a large step change and an average initial plasticity of zero, increasing the plasticity parameter (i.e., phenotypic variation due to plasticity) increased the probability of survival (Figure 1a, filled symbols), relative to no plasticity (*b *=* *0). Increasing the average initial plasticity caused a large increase in survival (Figure 1a, open symbols), even with a small plasticity parameter.

When plasticity was costly, the results for the survival probability were more complex and depended on the magnitude of the benefits of plasticity relative to its costs (Figure 1b). When the mean initial plasticity was zero, then a cost of plasticity caused a modest decrease in the probability of survival, relative to the same plasticity parameter with no cost (compare filled symbols in Figure 1a,b). In contrast, when the mean initial plasticity was 0.2 so that the population had a substantial fitness cost in the initial generations, and the plasticity parameter was small (0.2, 0.4) so that the benefits were weak, there was a large decrease in survival probability due to costs. In contrast, when the plasticity parameter was large (0.8, 1.0) so that the benefits were more substantial, there was only a modest decrease in the probability of survival due to the cost of plasticity. A pleiotropic correlation between plasticity and developmental noise had little effect on the survival probability (Figure 1c).

### 3.2 Step change – plasticity evolution

**Figure 4:**
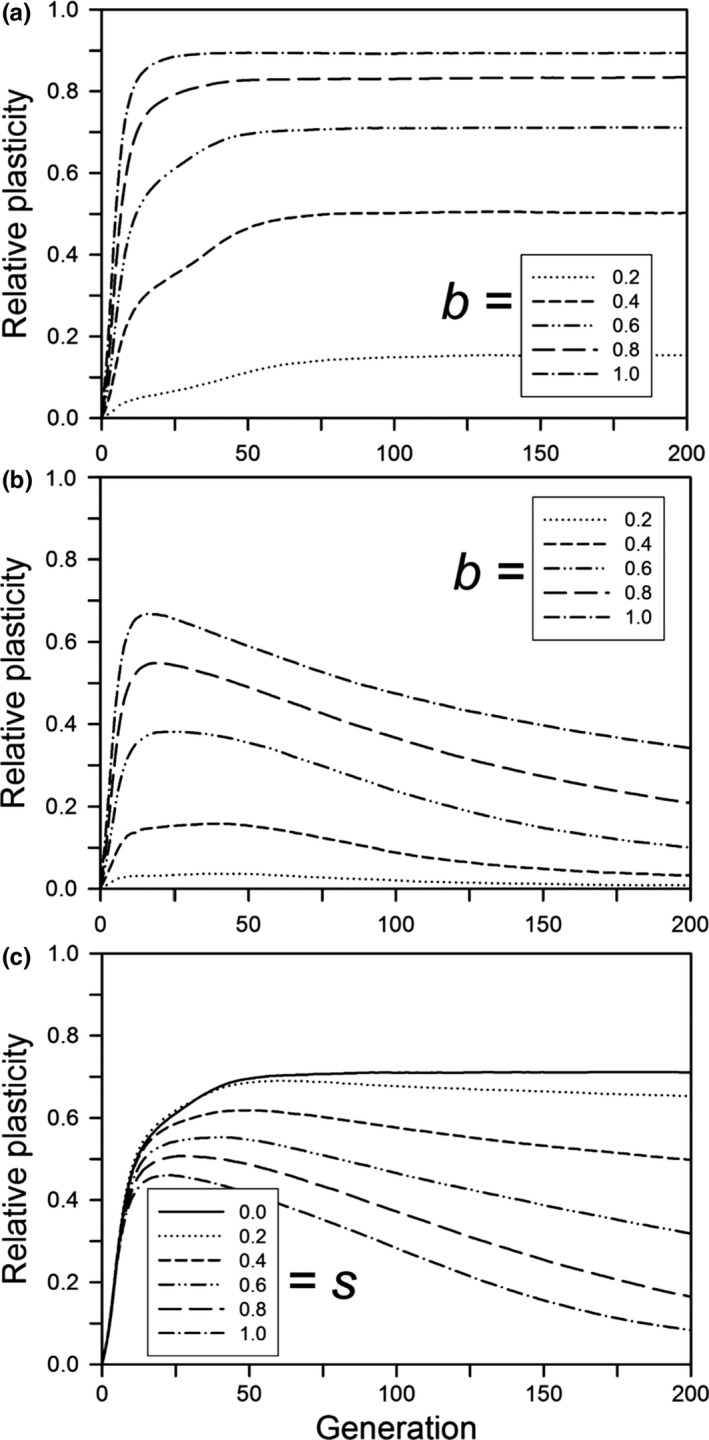
For a step change in the environment, the relative plasticity as a function of the time since the step change, for populations that survived for 500 generations, with no initial mean plasticity. Other parameters were as follows: *K *=* *256, *f *=* *4, ω = 1, *n = m = *10, μ = 0.0005, α^2^ = 0.05, ω_*P*_ = 1. (a) Without plasticity costs or developmental noise for different plasticity parameters (*b*); (b) with plasticity costs for different plasticity parameters (*b*) without developmental noise; (c) with developmental noise for different amounts of noise (*s*) without plasticity costs (*b *=* *0.6). The step change in the environment was 3.0 units

**Figure 5:**
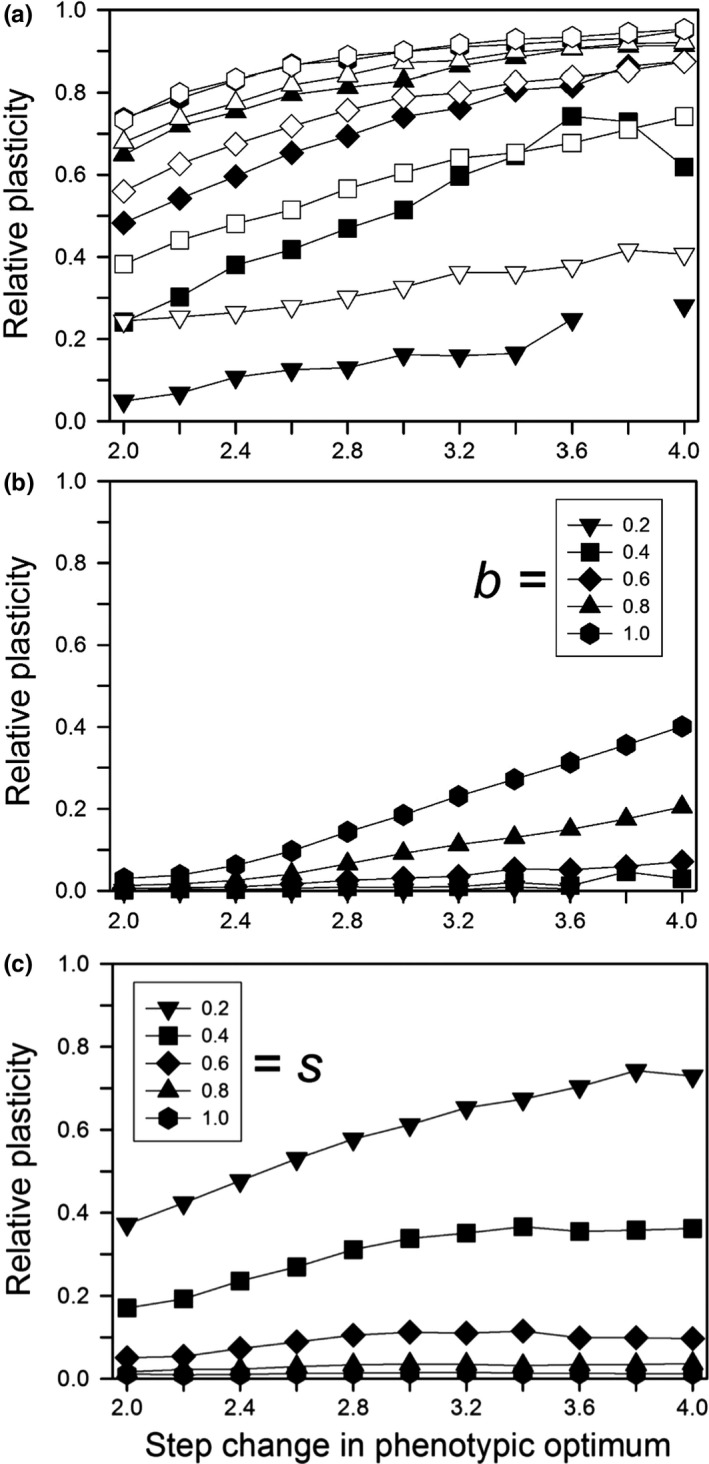
For a step change in the environment, the relative plasticity at generation 500 for different expected initial amounts of relative plasticity (0 = solid symbols; 0.2 = open symbols). Other parameters were as follows: *K *=* *256, *f *=* *4, ω = 1, *n = m = *10, μ = 0.0005, α^2^ = 0.05, ω_*P*_ = 1. (a) Without plasticity costs or developmental noise for different plasticity parameters (*b*); (b) with plasticity costs for different plasticity parameters (*b*) without developmental noise; (c) with developmental noise for different amounts of noise (*s*) without plasticity costs (*b *=* *0.6). When only solid symbols are shown, there was little or no difference without versus with initial plasticity. Missing symbols indicate that no population survived to 500 generations

The amount and pattern of plasticity evolution depended on the size of the step change and whether plasticity was costly. When the plasticity parameter was small, evolution to the new optimum occurred before plasticity had evolved much (Figure 4a, solid curve). This lack of plasticity evolution occurred for a small *b* even when the population had an initial average plasticity of 0.2 (Figure 5a). For large *b*, the amount of plasticity at 500 generations was about the same for equivalent values regardless of whether there was initial plasticity (Figure 5a). We note, however, that these results may be sensitive to departures from our assumptions of a linear reaction norm and Gaussian fitness function (Paenke, Sendhoff, & Kawecki, 2007).

**Figure 6:**
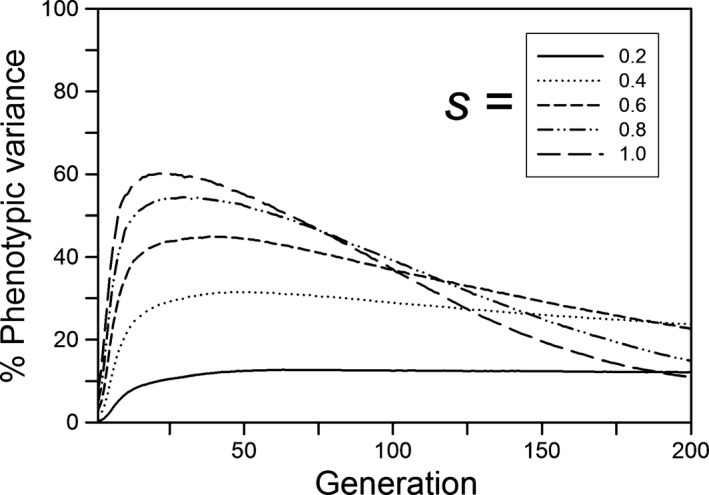
For a step change in the environment, the percentage of the phenotypic variation due to developmental noise caused by plasticity for different amounts of developmental noise (*s*) as a function of the time since the step change. The step change in the environment was 3.0 units, the plasticity parameter (*b*) was 0.6, and the expected value of initial plasticity was 0. Other parameters were as follows: *K *=* *256, *f *=* *4, ω = 1, *n = m = *10, μ = 0.0005, α^2^ = 0.05, ω_*P*_ **= 1

As expected, the greater the step change the greater the amount of plasticity at generation 500 (Figure 5). Whether or not plasticity was costly or was linked to developmental noise, the amount of plasticity rose quickly during the first 25 generations, except for the lowest plasticity parameter values (Figure 4). With no plasticity cost or limitation, the amount of plasticity then approached a constant value; only when costs were present or plasticity was linked to developmental noise did plasticity decline, albeit slowly. After 500 generations, plasticity was near zero (the mutation‐selection balance point) when plasticity was costly or linked to developmental noise except for large step changes when either the plasticity parameter (*b*) was large or developmental noise was low (Figure 5b,c), and we expect that those would approach zero with enough time. For plasticity linked to developmental noise, a substantial decrease in plasticity within the first 200 generations occurred only when the percentage of the phenotypic variance due to developmental noise rose to at least 40% (Figure 6). When plasticity was costly or linked to developmental noise, the initial amount of plasticity had a negligible effect on the amount of plasticity after 500 generations (results not shown).

**Figure 7:**
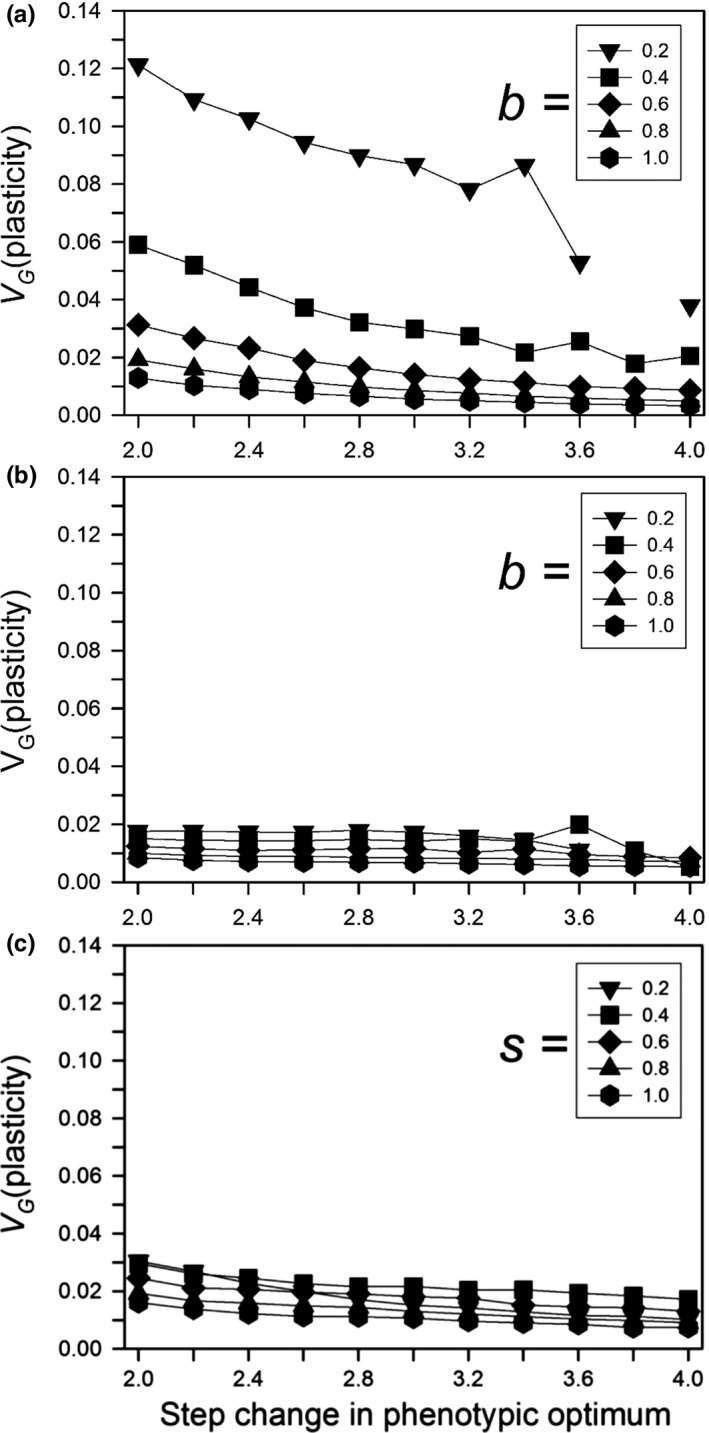
For a step change in the environment, the genetic variation for plasticity at generation 500 with no initial plasticity. Other parameters were as follows: *K *=* *256, *f *=* *4, ω = 1, *n = m = *10, μ = 0.0005, α^2^ = 0.05, ω_*P*_ = 1. (a) Without plasticity costs or developmental noise for different plasticity parameters (*b*); (b) with plasticity costs for different plasticity parameters (*b*) without developmental noise; (c) with developmental noise for different amounts of noise (*s*) without plasticity costs (*b *=* *0.6). There was little or no difference without versus with initial plasticity. Missing symbols indicate that no population survived to 500 generations

**Figure 8:**
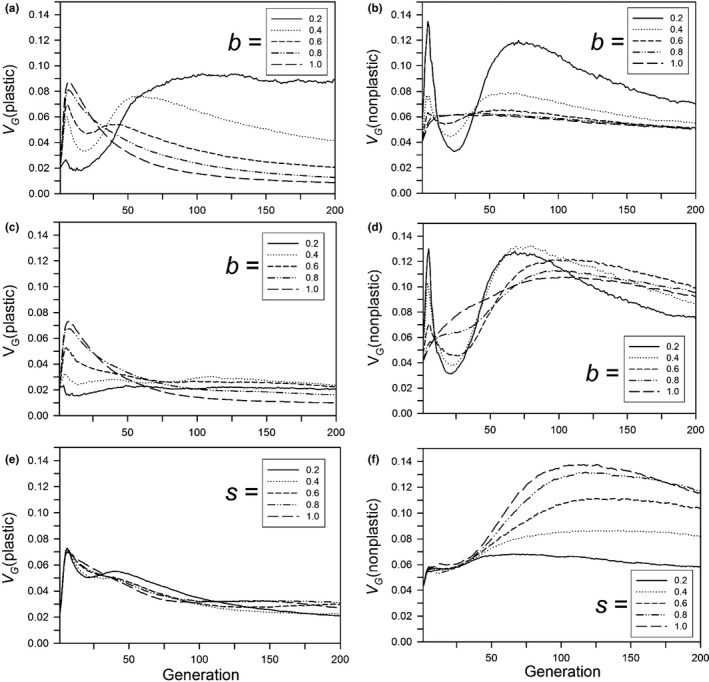
For a step change in the environment, the change in the genetic variation for the plastic (a, c, e) and nonplastic (b, d, f) variation as a function of the time since the step change, for populations that survived for 500 generations. Other parameters were as follows: *K *=* *256, *f *=* *4, ω = 1, *n = m = *10, μ = 0.0005, α^2^ = 0.05, ω_*P*_ = 1. (a, b) Without plasticity costs or developmental noise for different plasticity parameters (*b*); (c, d) with plasticity costs for different plasticity parameters (*b*) without developmental noise; (e, f) with developmental noise for different amounts of noise (*s*) without plasticity costs (*b *=* *0.6). The step change in the environment was 3.0 units

After 500 generations, the amount of genetic variation for plasticity (variance of the sum of plastic allelic values) had a simple relationship to the size of the step change and the plasticity parameter, but it had a complex temporal dynamic. After 500 generations, when plasticity was not costly or linked to developmental noise, the amount of genetic variation decreased with the size of the step change and the magnitude of the plasticity parameter (Figure 7a). When plasticity was costly or linked to developmental noise, the final amount of genetic variation was always low (Figure 7b,c). For its dynamics, if plasticity was not costly or linked to developmental noise and the plasticity parameter was large (0.8 or 1), the amount of genetic variation initially increased for a short period and then monotonically decreased, reaching a low level by 200 generations (Figure 8a). If the plasticity parameter was smaller, the initial rise was smaller and followed by a brief fall and then a rise, with a total change in magnitude of threefold to fourfold. The first rise is likely due to selection on the initial genetic variation, and corresponds to the initial fast increase in relative plasticity (Figure 4a). During this initial period, the population size dropped and the favorable alleles reached high frequency, causing the genetic variance to drop. However, for low values of *b*, the population was still far from the phenotypic optimum after this initial phase, and adaptation slowed as genetic variance declined (Figure 3a). If the population survived, its size then increased and new variation was generated, increasing the genetic variance (Figure 8a,b) and allowing adaptation to continue or even accelerate (Figure 3a). The result is that in the immediate generations after the step change in the environment, the amount of genetic variation for plasticity was positively correlated with the plasticity parameter, but after several hundred generations it was negatively correlated.

The same patterns held when there was a cost of plasticity (Figure 8c), although the peak variation was lower than without costs, especially for small *b*. In later generations, however, for small parameters the second increase in variation was much less with plasticity costs than without. For the genetic variation due to the nonplastic loci, the pattern was yet more complex with a similar threefold to fourfold change in magnitude (Figure 8b,d); in general the amount of variation and the amount of change in that variation was greater than that for plasticity if there were costs to plasticity or if *b* was small. In contrast, a limitation on plasticity had little effect on the amount of genetic variation for plasticity (Figure 8e) but resulted in higher amounts of genetic variation of the nonplastic loci, with about a 2‐fold change in magnitude through time (Figure 8f). The genetic correlation between the plastic and nonplastic loci was always small and negative, with the largest negative values (typically not more than −0.07) reached in the first few tens of generations; equilibrium values were approximately −0.01 (results not shown). Linkage disequilibrium between the two types of loci was therefore minor and transient.

### 3.3 Continual change – survival

**Figure 9:**
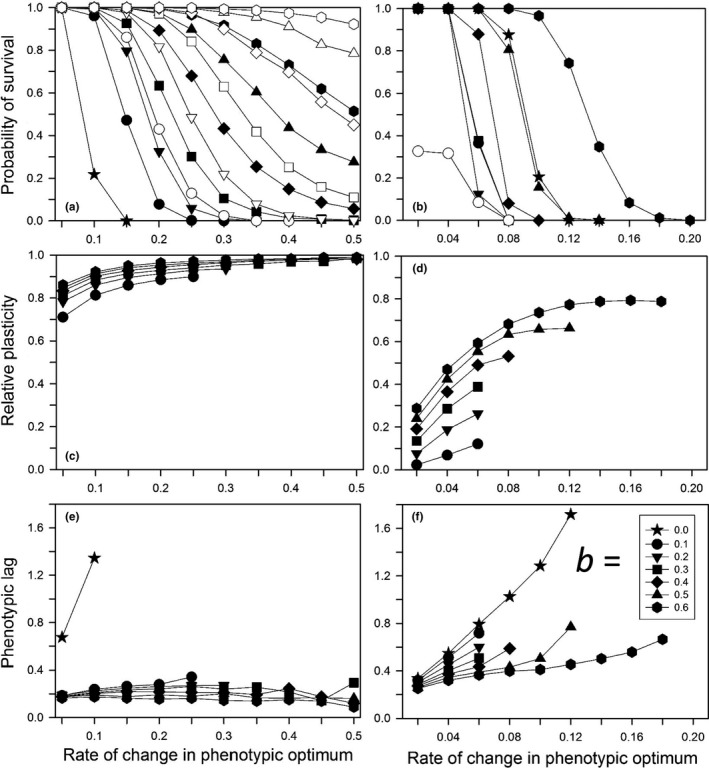
For continual environmental change, characteristics as a function of the per generation rate of change for different initial mean relative plasticities (0 = solid symbols; 0.2 = open symbols) and the plasticity parameter (*b*). Other parameters were as follows: *K *=* *256, *f *=* *4, ω = 1, *n = m = *10, μ = 0.0005, α^2^ = 0.05, ω_*P*_ = 1. (a) The probability of survival to generation 1,000 without plasticity costs; (b) the probability of survival with plasticity costs. (c) The relative plasticity at generation 1,000 (for populations that survived until then) without plasticity costs; (d) the final relative plasticity with plasticity costs. (e) The phenotypic lag at generation 1,000 (for populations that survived until then) without plasticity costs; (f) the phenotypic lag with plasticity costs. When only solid symbols are shown, there was little or no difference without versus with initial plasticity

For continual environmental change, when plasticity was absent (symbol = star), survival occurred only at low rates of change (Figure 9a,b). Plasticity costs had a bigger effect on survivorship with continual change than for a step change. When costs were absent, survivorship was close to or at 100% for rates of change below 0.2 units per generation, unless the plasticity parameter was small and the initial mean plasticity was 0. When costs were present, survivorship was 0% above 0.2 units. As a result we explored different rates of change for the two conditions (note different abscissa scales for left and right panels).

As with a step change, when plasticity was not costly the probability of survival increased as the plasticity parameter increased (i.e., phenotypic variation due to plasticity) and decreased with a greater rate of environmental change. Unlike the step change case, when plasticity was costly the probability of survival was not monotonic with the plasticity parameter (Figure 9b). At the smallest values (0.1–0.3), the survival probability was substantially lower than with no plasticity (*b *=* *0) because the cost of plasticity outweighed the benefit. As the plasticity parameter increased, survival increased, becoming greater than without plasticity at *b *=* *0.6, because now the benefit was greater than the cost.

One general difference from a step change was that with continual change the initial amount of plasticity was less important, as might be expected. In the absence of a cost of plasticity, having plasticity in the initial generation increased the probability of survival (Figure 9a); when plasticity was costly, those costs canceled the benefits of having initial plasticity so that survival differences were negligible, except for *b *=* *0.1 where survival was lower with initial plasticity again because the costs outweighed the benefits.

**Figure 10:**
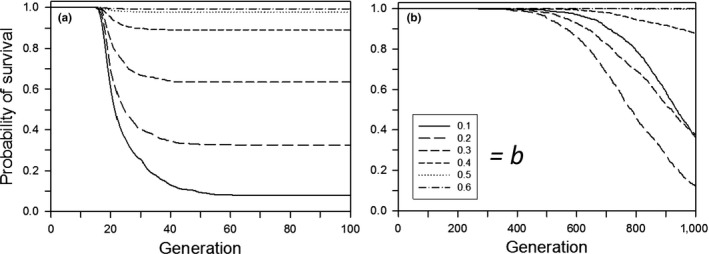
For continual environmental change, the probability of survival for different plasticity parameters (*b*) as a function of the time since the start of the change: (a) without plasticity costs and a rate of environmental change of 0.2 units/generation; (b) with plasticity costs and a rate of environmental change of 0.06 units/generation. The expected value of initial plasticity was 0. Other parameters were as follows: *K *=* *256, *f *=* *4, ω = 1, *n = m = *10, μ = 0.0005, α^2^ = 0.05, ω_*P*_ **= 1

**Figure 11:**
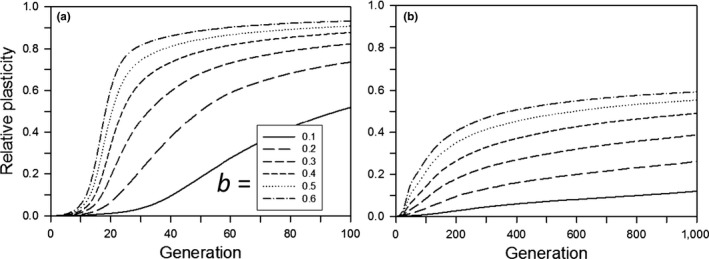
For continual environmental change, the change in the relative plasticity for different plasticity parameters (*b*) as a function of the time since the start of the change, for populations that survived for 1,000 generations: (a) without plasticity costs and a rate of environmental change of 0.2 units/generation; (b) with plasticity costs and a rate of environmental change of 0.06 units/generation. The expected value of initial plasticity was 0. Other parameters were as follows: *K *=* *256, *f *=* *4, ω = 1, *n = m = *10, μ = 0.0005, α^2^ = 0.05, ω_*P*_ **= 1

With no cost of plasticity, as with the step change, most extinctions occurred in the early generations (Figure 10a), although extinctions were earlier with the step change; in both cases there was a brief initial period with no extinctions. For conditions with low‐to‐moderate survivorship (*b *=* *0.1–0.3), those extinctions occurred before substantial plasticity evolved (Figure 11a) and most of the increase in plasticity occurred in the first 200 generations. In contrast, when plasticity was costly the amount of plasticity continued to increase for all 1,000 generations (Figure 11b). As a result, in later generations, there was an everincreasing fitness load leading to an increasing extinction rate (Figure 10b).

### 3.4 Continual change – plasticity evolution

The amount of plasticity at 1,000 generations was substantially affected by the cost of plasticity (Figure 9c,d). When plasticity was not costly, the relative plasticity was close to 1.0 for all but the lowest rates of change. In contrast, plasticity costs resulted in substantially lower amounts of plasticity and a mixed strategy of plastic and fixed genetic responses. Even for *b *=* *1.0, the relative plasticity was only 80% of the amount that would have permitted the populations to respond to environmental change by just a plastic change in phenotype.

### 3.5 Continual change – phenotypic lag

An important aspect of the evolutionary response to continual change is how far behind the optimum the population lags. A greater lag indicates a lower average fitness and a greater potential for extinction if the rate of change were to suddenly increase. When there was no cost of plasticity, the phenotypic lag was low regardless of the rate of change, which is consistent with the high relative plasticity (Figure 9e). That is, a pure plastic response by the population was sufficient to keep up with environmental change. Of course, this continual response is predicated on our assumption that there were no physiological or developmental limits to the plastic response. When there was a cost, the result was more complex (Figure 9f). Plasticity always decreased the lag relative to the no plasticity scenario. However, unlike in the no cost scenarios, the lag increased with the rate of change despite the increased amount of plasticity. The cost of plasticity was creating an additional fitness drag on the populations.

## 4 DISCUSSION

### 4.1 Genetic assimilation

Our results suggest that genetic assimilation as originally postulated by Waddington (1942) (namely, replacement of plasticity by nonplastic adaptation after a step change from one constant environment to another) will not occur or is unlikely to occur. If there is a step change in the environment and plasticity is not costly or linked to developmental noise, then there is no selection to replace the plastic determination of the trait with a fixed, nonplastic determination (Figure 4a), so genetic assimilation will not occur. If plasticity is costly or if it is linked to developmental noise, plasticity will be replaced by fixed genetic effects, but this replacement will likely take hundreds of generations (Figure 4b,c). This long period for the elimination of plasticity was also found by Lande (2009). In addition, although both Scheiner, Caplan, and Lyman (1991) and Tonsor, Elnaccash, and Scheiner (2013) found a genetic correlation between developmental noise and phenotypic plasticity, in both studies the magnitude of that effect was smaller than the amounts modeled here, again suggesting a very long elimination period. In most habitats, it is unlikely that the environment would remain stable for such an extended period, and any further change would result in new selection that favors plasticity. These conclusions also hold for West‐Eberhard (2003) expanded version of Waddington's theory. She broadened the factors that create a plastic response, but assumed the same subsequent dynamic.

In our model, we assumed that the cost of plasticity scaled with the genotypic value of plasticity (Equation (3)). Alternatively, we could have assumed that the cost of plasticity was fixed regardless of the amount of plasticity, which might occur if the cost was incurred from information gathering (e.g., the maintenance of sensory machinery or assessing predation). Depending on the assumed fixed cost, it could either be smaller or greater than the costs in our model, which would either increase or decrease the time to the elimination of plasticity. We could also have assumed that the cost of plasticity scaled with the phenotypic expression of the trait, such as if the cost for constructing a trait through a plastic developmental pathway was greater than that for a fixed developmental pathway. In that case, the rate of decline of plasticity might have been greater initially, but then slower as the phenotypic expression of the trait due to plasticity declined. So either alternative might have changed the specific evolutionary dynamics, but not the overall pattern or conclusion.

The requirement for plasticity to be costly or be linked to developmental noise adds another burden to the theory. In those cases, plasticity can be maintained in the initial population only through mutation. For the purposes of our model, we set the mutation rate to a relatively high value. The only estimate of the mutation rate for trait plasticity that we are aware of is that of Latta et al. (2015) for morphological and life history traits in *Daphnia pulex*. Mutational variation for plasticity was about half of the magnitude of that for the traits themselves. Thus, it is likely that mutation alone would be insufficient to maintain costly or limited plasticity in the initial population. Conversely, if the cost or limitation effects are unexpressed in the initial environment, mutation will allow the amount of (unexpressed) plasticity variation to increase unconstrained.

For trait plasticity to be replaced by fixed genetic effects there needs to be strong selection against plasticity. In the model of Lande (2009), plasticity is not replaced by fixed genetic effects. Rather, selection for a large plastic effect is transitory, with smaller amounts of plasticity being favored in the new environment because of limited environmental variation around a mean value. Similarly, plasticity exists in the initial population because of environmental variation, contra Waddington's theory which assumes a stable environment before and after the step change.

We are not aware of any demonstration of the process of genetic assimilation in a natural population. By this, we mean a strict interpretation of Waddington's theory entailing a step change between two stable environments. The closest to a demonstration that we are aware of are the papers of Aubret and Shine (2009, 2010) and Edgell, Lynch, Trussell, and Palmer (2009). Both show evidence of a decline in plasticity for populations with long associations with a novel prey or predator, respectively; however, neither study included measures of genetic variance, natural selection or environmental heterogeneity, so it is not possible to infer the actual evolutionary process. In an experimental test of genetic assimilation in *Caenorhabditis remanei* survival in response to heat shock (Sikkink, Reynolds, Ituarte, Cresko, & Phillips, 2014), plasticity for survival was substantially reduced at the selected temperature, but increased at higher and lower temperatures, which is inconsistent with the prediction of genetic assimilation of a general decrease in plasticity. Even Waddington's (1953, 1956) experiments are open to an alternative interpretation. He selected on categorical (i.e., binary) morphological traits in *Drosophila melanogaster* that showed a threshold plastic response to rearing temperature. In all cases, he measured the plasticity of the trait in only two environments and interpreted a change in the phenotype expressed in the original environment as a change in trait plasticity. However, there might not have been any change in the parameter(s) of the reaction norm. Instead, evolution might have occurred on the elevation of the reaction norm, in our parlance selection on the nonplastic loci. Only by measuring the reaction norm over multiple environments, including those intermediate to and outside the range of the two selective environments, can the evolution of reaction norm on a threshold response be assessed.

Thus, despite the wide acceptance of genetic assimilation as a mechanism as evidenced by the hundreds of times that Waddington (1953) has been cited, the mechanism has not been demonstrated to be important. We emphasize, though, that we are not discounting the importance of phenotypic plasticity in the response to environmental change. Rather, we doubt that trait plasticity will be replaced by fixed genetic effects, unless the selective environment is very stable, or unless novel costs to plasticity emerge after the environmental change. To test whether genetic assimilation has occurred in nature, it is necessary to show both that trait plasticity is transient and that the decline in plasticity is due to a cost or limitation of plasticity and not to other possible selective factors.

Also worth noting in the results (e.g., Figure 4) is that if plasticity is selected due to an abrupt environmental change, then there can be a transient plasticity that gradually wanes, but nonetheless lingers for many generations. So, sporadic bouts of environmental change can lead to genetic signatures of plasticity in populations well outside those episodes of change.

### 4.2 The Baldwin effect

Trait plasticity as an enhancement to continual evolutionary change as originally postulated by Baldwin (1896) may be an important evolutionary mechanism. If plasticity is not costly, then trait plasticity is highly favored (Figure 9c). This result is similar to the result found for temporal variation around a central mean (e.g., Lande, 2009; Scheiner, 2013) and for spatial environmental variation (Scheiner & Holt, 2012). However, if plasticity is costly, plasticity will generally be disfavored unless the rate of change is great enough that the costs of plasticity do not outweigh its benefits (Figure 9b). Even then, evolution favors a mix of plastic and fixed genetic effects (Figure 9d). A similar interaction effect of the rate of change with the cost of plasticity was found by Chevin et al. (2010, fig. 2), and Scheiner (2014) similarly found strong selection against plasticity when it was coupled with developmental noise.

Our model assumed that there were no limits to the magnitude of the plastic response. This is unlikely to be true in nature; a mouse will not likely grow to the size of an elephant as a plastic response, or even the size of a St. Bernard, no matter what environment you put it in. The Baldwin effect will be constrained by the biology of the organism. But it is also true that continual, unidirectional changes of the magnitude that we modeled are unlikely to occur indefinitely. High rates of change may happen over short periods of time, but will be mixed with slower directional change or periods of reversal. For example, global temperatures are increasing overall, but fluctuate from year to year, particularly at the local level where selection is manifest.

### 4.3 Evolutionary rescue

Whether or not plasticity helps or hinders evolutionary rescue following a step change in the environment depends on whether plasticity is costly. When plasticity is not costly (even if it is linked to developmental noise), it always helps evolutionary rescue (Figure 1a,c), especially if the mean plasticity in the population is nonzero prior to the environmental change (Chevin & Lande, 2010). The effect of plasticity is dramatically different; however, if plasticity is costly. Costly plasticity hindered evolutionary rescue when the amount of environmental change was small such that the benefits of plasticity did not outweigh its costs (Figure 1b).

For continual environmental change, the evolutionary process is somewhat different than that for evolutionary rescue following an abrupt change. With evolutionary rescue after a step change there is an initial period of maladaptation and a decline in population size until adaptive evolution leads to positive population growth. With continual change, the population may initially be reasonably well adapted to the environment, but as the magnitude of the change becomes greater, maladaptation increases as the population trait value lags behind the moving optimum. Our base model without plasticity resulted in 100% extinction at relatively low rates of environmental change (Figure 9a,b), consistent with quantitative genetic models (Bürger & Lynch, 1995; Kopp & Matuszewski, 2014). In our model, noncostly plasticity allowed the population to keep pace with the environmental change. However, costly plasticity led to an increasing fitness drag and an increasing chance of extinction, except for the lowest rates of change (Figure 10b, see also Chevin et al., 2010; Nunney, 2015). Alternative assumptions about the form of plasticity costs may have changed the details of these effects, but likely would not have altered the overall conclusions.

Thus, for the two types of changes – step and continual – the magnitude of environmental change had opposite effects in combination with plasticity costs. For a step change, a greater one‐time change meant that the benefits of plasticity outweighed its costs. For continual change, a greater rate of change increased the fitness drag due to plasticity costs. How these two cost effects might play out for more complicated patterns of environmental change is unclear.

### 4.4 Response to climate change

Changes in the global climate, along with other human‐caused environmental alterations, are having profound effects on Earth's biota. One class of effects is changes in patterns of trait selection. Many questions have been raised about what sorts of evolutionary responses will occur (Hoffmann & Sgrò, 2011; Merilä & Hendry, 2014; Valladares et al., 2000; Visser, 2008), with phenotypic plasticity sometimes being put forward as a panacea to climate change challenges (Matesanz, Gianoli, & Valladares, 2010; Nicotra et al., 2010). Our results provide a cautionary note to those expectations. Plasticity can enhance adaptation and prevent extinction under some conditions, but not others (Paenke et al., 2007). Our models indicate the critical information needed to assess its potential role: the future rate and pattern of change of trait optima and the costs and phenotypic limits of trait plasticity.

The environment is changing, but that change varies in both rate and direction. Nor is there a simple, linear relationship between a change in the environment and a change in optimal trait value. We need information on likely patterns of environmental change and how they will affect trait plasticities and fitness, meaning that all components of the process must be carefully measured. Our model did not consider variation in the rate or direction of change. Such variation can increase selection for plasticity, but that result strongly depends on the pattern of variation (Scheiner, 2013). As shown by Lande (2009), selection that favors plasticity prior to a large change in the environment can foster an evolutionary response that includes plasticity. Similarly, a plastic response to directional change can be enhanced if the pattern of short‐term, nondirectional change also favors plasticity. Not all short‐term variation will favor plasticity, so studies of current selection for plasticity may help predict the future role of phenotypic plasticity.

A key parameter in our model was whether plasticity was costly. There is little evidence for plasticity costs in multicellular eukaryotes (Murren et al., 2015) except under very stressful conditions (Van Buskirk & Steiner, 2009). On the other hand, a recent meta‐analysis found that adaptive phenotypic plasticity is less common in plants than expected – only 33% of traits showed adaptive plasticity (Palacio‐López, Beckage, Scheiner, & Molofsky, 2015). Costs of plasticity are frequently invoked in models to constrain plasticity evolution (e.g., Botero, Weissing, Wright, & Rubenstein, 2015; Fischer, Doorn, Dieckmann, & Taborsky, 2014; Lande, 2014; Sultan & Spencer, 2002), and they could explain its less‐than‐expected frequency, but not if such costs are absent. One way to reconcile these observations may be that costly plasticity is continually being selected against so that the only extant plasticity is for traits where costs are minimal within the range of currently expressed plasticity. However, if long‐term climate change pushes organisms to express much more extreme values of plasticity, costs may manifest. Information on the potential for such unexpressed, latent costs is needed.

### 4.5 The evolution of plasticity

We can study the process of adaptation to environmental heterogeneity at two levels that of the entire organism and that of individual traits. In advocating for the importance of phenotypic plasticity in evolution, Bradshaw (1965) distinguished between these two levels by referring to the phenotypic flexibility of the organism versus the phenotypic plasticity of the trait (Peirson, 2015). We encourage this terminology as it clarifies the distinction between the fitness outcome (e.g., a wide environmental tolerance) and the phenotypic cause (e.g., phenotypic plasticity in specific traits). It also makes explicit the possibility that environmental tolerance can result from different causes: phenotypic plasticity versus a jack‐of‐all‐trades fixed phenotype versus adaptation by genetic specialization via genetic polymorphism.

Our model considered selection on a single trait. But the environmental tolerance of an organism results from the interactions of all parts of the phenotype, interactions which might themselves depend upon environmental conditions. Such interactions might be important for long‐term patterns of adaption. Plasticities of different traits can be genetically correlated (e.g., Scheiner et al., 1991; Schlichting, 1989). Similarly, the “environment” is not a unitary factor. Climate change involves multiple factors (e.g., mean temperatures, maximal temperatures, total precipitation, seasonality of precipitation) that can vary independently and have different effects on trait selection and organismal fitness. Samani and Bell (2016) found that selection for plasticity in response to one stress factor increased plasticity to other stress factors. Thus, multiple, varying environmental factors might enhance selection for trait plasticity and the overall plastic response to environmental change.

## AUTHOR CONTRIBUTIONS

All authors contributed to the development of the ideas and the writing of the manuscript. The models were coded and run by MB.

## CONFLICT OF INTEREST

None declared.
